# Peptides: Emerging Candidates for the Prevention and Treatment of Skin Senescence: A Review

**DOI:** 10.3390/biom15010088

**Published:** 2025-01-09

**Authors:** Andrada Pintea, Andrei Manea, Cezara Pintea, Robert-Alexandru Vlad, Magdalena Bîrsan, Paula Antonoaea, Emöke Margit Rédai, Adriana Ciurba

**Affiliations:** 1 Medicine and Pharmacy Doctoral School, George Emil Palade University of Medicine, Pharmacy, Science, and Technology of Targu Mures, 540142 Targu Mures, Romania; 2 Pharmaceutical Technology and Cosmetology Department, Faculty of Pharmacy, George Emil Palade University of Medicine, Pharmacy, Science and Technology of Targu Mures, 540142 Targu Mures, Romaniaadriana.ciurba@umfst.ro (A.C.); 3 Department of Drug Industry and Pharmaceutical Biotechnology, “Grigore T. Popa” University of Medicine and Pharmacy, 700115 Iasi, Romania

**Keywords:** cosmetic peptides, anti-aging products, permeability, nano-systems

## Abstract

One class of cosmetic compounds that have raised interest of many experts is peptides. The search for ingredients with good biocompatibility and bioactivity has led to the use of peptides in cosmetic products. Peptides are novel active ingredients that improve collagen synthesis, enhance skin cell proliferation, or decrease inflammation. Based on their mechanism of action, they can be classified into signal peptides, carrier peptides, neurotransmitter inhibitor peptides, and enzyme inhibitor peptides. This review focuses on the main types of peptides and their application in the cosmetic field, underlining their main limitations. One of the most significant drawbacks of cosmetic peptides is their poor permeability through membranes, which limits their delivery and effectiveness. As a result, this review follows the methods used for improving permeability through the stratum corneum. Increasing peptide bioavailability and stability for enhanced delivery to the desired site of action and visible effects have become central points for the latest research due to their promising features. For this purpose, several methods have been identified and described. Physical techniques include thermal ablation (radiofrequency and laser), electrical methods (electroporation, iontophoresis), mechanical approach (microneedles), and ultrasounds. As an alternative, innovative formulations have been developed in nano-systems such as liposomes, niosomes, ethosomes, nanoemulsions, and other nanomaterials to reduce skin irritation and improve product effectiveness. The purpose of this review is to provide the latest information regarding these noteworthy molecules and the reasoning behind their use in cosmetic formulations.

## 1. Introduction

Recently, the cosmetic industry has seen impressive development due to a better understanding of the skin’s physiological mechanisms and a growing demand for innovative products. This has led to the expansion of new research techniques, novel active ingredients, and vehicles, changing our understanding of how cosmetics influence and improve skin conditions. These developments have propelled cosmetics into a new era of scientifically engineered products for the development of a wide range of skin phenotypes and affections. Originally conceived as preparations intended to improve personal appearance by direct application to the skin, cosmetics have now taken a new role in dermatology by supporting the management of many skin conditions [1].

The aspect of normal and healthy-looking skin reflects flexibility, elasticity, hydration, and firmness. All of these characteristics are influenced by the consistency of collagen and elastin fibers, the keratinization of the stratum corneum, the degree of hydration, and the water-binding capacity [2]. However, due to intrinsic and extrinsic factors, the skin is subject to degradation and displays signs of aging. Exposure to UV radiation will change the structure of the skin by affecting the structural proteins, leading to loss of moisture, firmness, elasticity, softness, and the appearance of spots and wrinkles. This process is known as photoaging and is one of the main causes of accelerated skin aging [3].

Collagen is a fibrillar protein that dominates the extracellular matrix of most connective tissues and is responsible for the structure and biochemical properties of the skin. The most common type of collagen found in organisms is type I [4]. The process of aging is associated with decreased collagen synthesis and increased cleavage of fibrils but also exacerbated levels of matrix metalloproteinases, whose purpose is to break down collagen and elastin fibers [5].

Due to its important aesthetic function, the skin has captivated people’s interest since the beginning of time. Treating wrinkles and aging signs using cosmetic products has become a widely used practice. Many products are intended to improve the nature of deteriorated collagen and firmness or paralyze the muscles that cause wrinkles. The active ingredients used in modern anti-aging cosmetics have additional effects, exhibiting more complex functions (protection, nourishing, moisturizing, exfoliating) [6].

Bioactive peptides, usually containing 3–30 amino acids (AA), are natural or synthetic compounds involved in a variety of physiological activities such as cellular protection, thermoregulation, and immunity. Peptides exhibit many biological properties, including antioxidant, antimicrobial, and anti-inflammatory activities, in addition to their properties of inhibiting aging-related enzymes [7].

Over the last decades, the study of peptides has extended to the field of cosmetics. Their applications can vary from enhancing skin cell proliferation, reduction in pigmentation spots, decreasing inflammation, and improving skin barrier functions.

The most common classification of cosmetic peptides is based on their mechanism of action as follows: signal peptides, carrier peptides, neurotransmitter inhibitor peptides, and enzyme inhibitor peptides (Table 1) [4].

The amino acid sequence of a cosmetic peptide plays a crucial role in determining its effects on the skin. Each amino acid in a peptide sequence contributes to the shape and charge of the molecule, therefore determining how the peptide interacts with the receptors and enzymes and how it diffuses through the lipid layer. In a study focused on understanding how the amino acid sequence affects the interaction of peptides with lipid membranes, it has been observed that peptides with a positive charge bind more easily to the hydrophobic layer than neutral or negatively charged peptide molecules. This study has highlighted that amino acids at various positions in the peptide sequence bind differently to the membrane. Considering this, peptides containing amino acids with a positive charge, such as lysine, can bind with a higher frequency to the membrane if they are located at the extremities of the sequence. Nevertheless, peptides composed of less hydrophobic, non-polar residues (such as valine and alanine) are much less likely to adsorb to the membrane than phenylalanine peptides [8]. Liu et al. have studied the adsorption of four similar peptides containing the same amino acids but arranged differently in the sequence. It was revealed that the contribution of each amino acid to the peptide’s hydrophobicity was affected by the position of the amino acid. When placed in the middle of the chain, the most lipophilic amino acid showed the highest hydrophobicity [9]. By modifying sequence length, amino acid composition, and structural modifications, peptide developers can create targeted, stable, and efficient skin-care ingredients with specific actions.

**Table 1:** Cosmetic peptides.

Type of Peptide	Peptide Name	Commercial Name	Amino Acid Sequence	Molecular Weight (g/mol)	Role	Reference
Signal peptides	Palmitoyl Pentapetide-4	Matrixyl^®^	Lys-Thr-Thr-Lys-Ser	802.1	Stimulates the production of collagen and other extracellular matrix components.	[10]
	Tripeptide-10 Citrulline	Decorinyl™	Lys-Asp-Ile	530.6	Regulates the fibrillogenesis process and collagen fibril dimensions.	[11]
	Palmitoyl Hexapeptide-12	Biopeptide El™	Val-Gly-Val-Ala-Pro-Gly	737.0	Promotes collagen and elastin production. Anti-inflammatory effect.	[12]
	Palmitoyl Tripeptide-5	Syn^®^-Coll	Lys-Val-Lys	611.9	Boosts collagen production by stimulating TGF-β production and increasing cellular communication.	[13]
	Palmitoyl Tripeptide-1	Biopeptide CL™	Gly-His-Lys	578.8	Stimulates collagen and glycosaminoglycans.	[14]
	Acetyl Tetrapeptide 9	Dermican™	*N*-Acetyl-Gln-Asp-Val-His	539.5	Stimulates collagen type I and lumican synthesis.	[12]
	Acetyl Tetrapeptide-11	Syniorage™	N-Acetyl-Pro-Pro-Tyr-Leu	530.6	Accelerates keratinocyte growth and syndecan-1 synthesis.	[12]
	Elaidoyl Tripeptide-37	Lipospondin	Elaidyl-Lys-Phe-Lys-OH	578.79	Stimulates TGF-β, promoting collagen and elastin production.	[15]
	Peptamide-6 /Hexapeptide-11	ND	Phe–Val–Ala–Pro–Phe–Pro	676.8	Modulates key genes responsible for collagen synthesis, improving skin elasticity.	[16]
	Trifluoroacetyl Tripeptide-2	Progeline^TM^	TFA-Val-Tyr-Val	475.46	Inhibits progerin synthesis, a cell-aging accelerator protein.	[17]
	Palmitoyl Tetrapeptide-7	Rigin™	Gly-Glu-Pro-Arg	694.9	Decreases IL-6 secretion, reduces inflammation after UVB exposure, and stimulates laminins IV and V as well as collagen VII production.	[18]
	Heptapeptide	Perfection Peptide P7™	Ac-Asp-Glu-Glu-Thr-Gly-Glu-Phe-OH	867.8	Protects skin cells against UV damage by stimulating Nrf2-dependant antioxidant enzymes.	[19]
	Tetrapeptide-30	PKEK	Pro-Lys-Glu-Lys	500.6	Reduces hyperpigmentation by inhibiting the tyrosinase enzyme and blocking the transfer of melanin.	[20]
	Tetrapeptide-21	GEKG	Gly-Glu-Lys-Gly	389.40	Stimulates collagen synthesis at the mRNA and protein levels while also positively influencing hyaluronan and fibronectin.	[21]
	SA 1-III	KP1	Ac-Met-Gly-Lys-Val-Val-Asn-Pro-Thr-Gln-Lys-NH2	1142.4	Modulates collagen turnover.	[22]
Carrier peptides	Copper Tripeptide-1	Cu-GHK	Gly-His-Lys	400.90	Modulates matrix metalloproteinase expression, accelerates skin regeneration, reduces inflammation, promotes the production of collagen and glycosaminoglycans, and has antioxidant properties.	[23]
	Manganese Tripeptide-1	GHK-Mn^2+^	Gly-His-Lys	392.29	Reduces hyperpigmentation and has antioxidant properties.	[23]
Neurotransmitter inhibitor peptide	Acetyl Hexapeptide-3	Argirelin^®^	Glu-Glu-Met-Gln-Arg-Arg	887.0	Inhibits neurotransmitter release and muscle contraction.	[24]
	Pentapeptide-18	Leuphasyl^®^	Tyr-Ala-Gly-Phe-Leu	569.6	Modulates the release of certain neurotransmitters.	[25]
	Tripeptide-3	SYN-AKE^®^	Ala-Pro-Dab	495.6	Acetylcholine receptor antagonist.	[14]
	Acetyl octapeptide-1/3	SNAP-8™	Glu-Glu-Met-Gln-Arg-Arg-Ala-Asp	1073.2	Blocks the SNARE complex by inhibiting SNAP-8.	[26]
	Acetyl Dipeptide-1 Cetyl Ester	Calmosensine™	Tyr-Arg	603.8	Stimulates the skin nerve cells to release met-enkephalin, resulting in muscle relaxation and reduced inflammation.	[27]
	Palmitoyl Tripeptide-8	Neutrazen™	His-Phe-Arg	695.9	Reduces the production of UVB-induced inflammatory cytokines such as IL-8.	[18]
	Acetyl Tetrapeptide-15	Skinasensyl™	Tyr-Pro-Phe-Phe	613.7	It limits the release of pro-inflammatory neuromediators and reduces skin hyperreactivity.	[18]
	Pentapeptide 3	Vialox^®^	Gly-Pro-Arg-Pro-Ala	495.6	Reduces muscle cell contraction by acting as a competitive antagonist at the acetylcholine postsynaptic membrane receptor.	[28]
Enzyme inhibitor peptide	Soybean peptides	ND	3-6 amino acids from soybean	-	Neutralize free radicals, increase collagen synthesis, offer UV protection.	[29]
	Rice peptides	ND	Obtained from the silk gland of the silkworm *Bombyx mori*	-	Antioxidant and anti-inflammatory effects.	[30]
	Silk peptides	ND	Obtained from rice protein	-	Inhibit the activity of metalloproteinases and stimulate the synthesis of hyaluronan synthase 2 gene.	[31]

Lys—Lysine; Thr—Threonine; Ser—Serine, Asp—Aspartic acid; Ile—Isoleucine; Val—Valine; Gly—Glycine; Ala—Alanine; Pro—Proline; His—Histidine; Gln—Glutamine; Tyr—Tyrosine; Leu—Leucine; Phe—Phenylalanine; Glu—Glutamic acid; Thr—Threonine; Met—Methionine; Arg—Arginine; Dab—Diaminobutyroyl Benzylamide Diacetate; ND—non-defined.

## 2. Applications of Cosmetic Peptides

**Figure 1:**
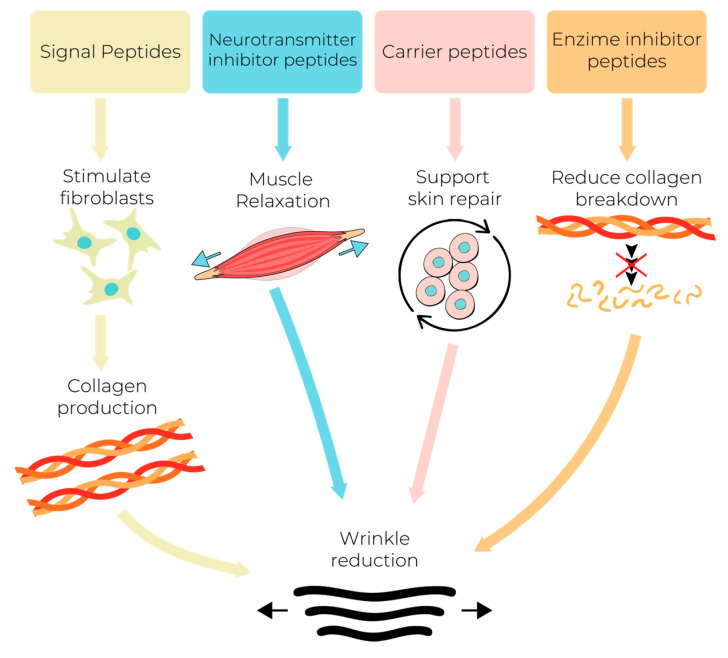
Anti-wrinkle mechanism.

Cosmetic peptides have emerged as powerful, innovative tools in modern skincare, offering solutions to a variety of skin concerns. They have become remarkable compounds due to their main purpose as anti-wrinkle agents. Cosmetic peptides reduce existing wrinkles and prevent new ones from forming through a variety of mechanisms of action, which can act synergically for enhanced results. Signal peptides are used to stimulate fibroblast cells to increase collagen production and improve skin elasticity; neurotransmitter inhibitor peptides relax facial muscles, minimizing expression wrinkles; carrier peptides deliver essential trace elements to support skin repair, and enzyme inhibitor peptides reduce collagen breakdown, preserving the integrity of the skin (Figure 1) [32,33].

One of their most significant effects is their ability to enhance the stimulation of collagen production. This property is mostly linked to signal peptides based on the capacity to mimic the transmitted signal during the synthesis of extracellular matrix proteins, such as collagen and elastin, which results in increased fibroblast activity, with consequent production of collagen and elastin. Peptides are used to restore collagen levels affected by fibroblast senescence, which slows their proliferation and reduces collagen synthesis (especially type I) while also addressing its degradation by proteolytic enzymes to achieve more elastic, firm, and smoother skin [34]. Additionally, they have metabolic functions and can act as growth factors, such as in the case of Hexapeptide-12, a signal peptide that is able to activate protein kinase C, an enzyme responsible for cell growth and development [35].

Recent research has discovered that some cosmetic peptides possess wound healing and tissue regeneration properties by promoting tissue repair, modulating inflammation, and enhancing cellular communication. One of the most notable compounds from this class is Copper Tripeptide-1, a carrier peptide that stabilizes and delivers trace elements such as copper or manganese necessary for wound healing and various enzymatic processes [36]. It was first described by Pikard et al. as a growth factor for mature cells, but other effects have since been reported. GHK–Cu works as a powerful chemotactic agent for monocytes, macrophages, and mast cells. It promotes nerve tissue regeneration, stimulates angiogenesis in vivo, ensuring a steady supply of oxygen and nutrients to the healing site, and enhances the expression of extracellular matrix components in both in vivo and in vitro settings, rebuilding damaged tissue [37]. The peptide sequence of GHK–Cu is found in the proteins of the extracellular matrix, such as the α chain of human collagen, indicating that it may be released during collagen production at the site of the wound [38]. This carrier peptide plays a crucial role in wound healing by delivering copper, which is essential for metalloproteinase activity [32]. Siméon et al. have proved that repeated injections of glycyl–histidyl-lysine-Cu^2+^ (2 mg/inj.) stimulate wound tissue production accompanied by type I collagen and glycosaminoglycans proliferation [39]. The targeted action of cosmetic peptides on skin cells complements and, in some cases, enhances the effects of other ingredients like copper sulfate, zinc sulfate, hyaluronic acid, or vitamin E. They are often combined with traditional actives to create a synergic effect. For example, carrier peptides can be combined with hyaluronic acid to facilitate hydration while promoting skin repair [40]. Cosmetic peptides can also be combined with zinc sulfate for better antimicrobial effect and lesion-healing and with vitamin E in antioxidant formulations, addressing both structural and oxidative damage [41]. While the traditional ingredients perform specific roles, peptides enhance and complement these effects, making them a versatile and powerful addition to modern wound-healing formulations.

Furthermore, several peptides can also possess antioxidant properties, counteracting the excessive production of reactive oxygen species (ROS). Different factors such as UV radiation, toxins, inflammation, or other exogenous or endogenous agents can intensify its negative effects. Peptides contribute to the reduction in oxidative stress in the skin by scavenging free radicals through different pathways, resulting in delaying the skin’s aging process [42]. The antioxidant capacity of peptides can be predicted by investigating their structure–activity relationships, molecular weight, amino acid composition, and sequence. Low-molecular-weight peptides (<1 kDa) can substantially enhance antioxidant activity, likely due to their ability to act as electron donors, neutralizing free radicals and preventing chain reactions. The antioxidant effect has been previously linked to specific amino acids: Trp, Phe, and His act as hydrogen donors and improve ROS scavenging capacity; Val, Leu, and Tyr inhibit lipid peroxidation and enhance the accessibility of fat-soluble ROS or polyunsaturated fatty acids [43]. A study conducted by Uno et al. has highlighted that the presence of aromatic amino acids at the C-terminus or the hydrophobicity of N-terminal residues could improve the antioxidant effects of peptides [44].

## 3. Classification of Cosmetic Peptides

### 3.1. Signal Peptides

**Figure 2:**
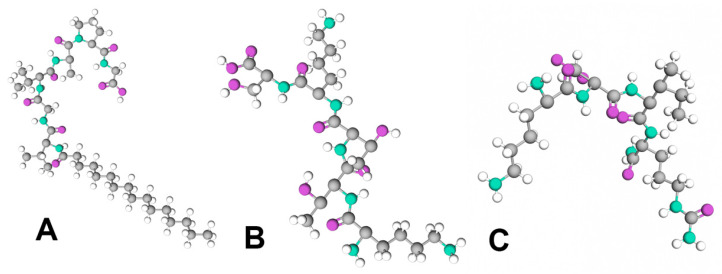
Signal peptides: (**A**) Palmitoyl Hexapeptide-12; (**B**) Tripeptide-10 Citrulline; (**C**) Palmitoyl Pentapeptide-4.

Signal peptides are active ingredients used in cosmetic products due to their capacity to stimulate skin fibroblasts, leading to increased production of collagen, elastin, fibronectin, glycosaminoglycan, and proteoglycan. This is possibly due to their ability to modulate the protein turnover of the skin, activating intracellular signaling pathways, for example, transforming the growth factor–beta pathway, thus promoting fibroblast proliferation and the upregulation of genes responsible for collagen and elastin synthesis [45]. Additionally, they can activate protein kinase C, an enzyme responsible for cell growth and migration [13]. This class of peptides has a three-part structure: a positively charged amino-terminal part; a hydrophobic central part; and a polar carboxyl-terminal part. Signal peptides are highly significant for their ability to open protein channels, enabling the translocation of synthesized proteins to their targeted sites of action [46]. Signal peptides (Figure 2) include Palmitoyl Pentapetide-4, Tripeptide-10 Citrulline, and Palmitoyl Hexapeptide-12, which will be further described.

#### 3.1.1. Palmitoyl Pentapetide-4

Palmitoyl Pentapeptide-4 (Matrixyl^®^) acts as a signaling agent after advancing through the stratum corneum into the dermis. There, it binds to the receptor situated on the cell surface, and through a series of cellular pathways, it boosts collagen types I and III production. Moreover, this peptide can intervene with collagen degradation, resulting in higher concentrations of fibrillar proteins [10]. Palmitoyl Pentapeptide-4 can accelerate procollagen production and regulate hyaluronic acid synthesis in fibroblasts [47]. This peptide works as a collagen stimulator, leading to improved skin elasticity, smoother and firmer texture, and visible reduction in fine lines and wrinkles. This active ingredient in a concentration of 3% has been proven to be safe, non-irritating, and non-sensitizing, tolerated by all skin types, including oily and acne-prone skin [48]. Robinson et al. have conducted a double-blind, placebo, randomized study on ninety-three Caucasian female subjects presenting facial wrinkles and fine lines. After the 12-week trial period of applying oil in water cream containing Palmitoyl Pentapeptide-4, expert graders identified an improvement in fine lines and overall skin appearance [49].

#### 3.1.2. Tripeptide-10 Citrulline

Tripeptide-10 Citrulline is a tetrapeptide with the amino acid sequence (lysine–asparagine–isoleucine–citrulline), which mimics the structure of decorin. Decorin is a protein that binds to collagen fibrils and controls collagen genesis, an essential process in tissue formation, consequently managing fibril dimensions, diameter, and spacing [50]. In vitro tests have shown that Tripeptide-10 Citrulline can target collagen fiber organization, making them thinner and more uniform, improving their overall cohesion [51].

#### 3.1.3. Palmitoyl Hexapeptide-12

Palmitoyl Hexapeptide-12 is a peptide that reduces the production of pro-inflammatory cytokines by keratinocytes and fibroblasts, such as interleukine-6 (IL-6). This type of cytokine is usually found in low levels but can be released together with reactive oxidative species (ROS) after UV aggression. ROS can alter the integrity of the skin matrix. Considering this, it is believed that Palmitoyl Hexapeptide-12, through its mechanism, can prevent the degradation of the skin and maintain its integrity [34].

### 3.2. Carrier Peptides

The main role of carrier peptides is to transport oligo-elements such as copper and manganese to the skin to be used by epithelial cells in physiological activities. One of the metals most often transported by this type of peptide is copper, which is involved in wound-healing processes, collagen synthesis, melanogenesis, and superoxide dismutation, having antioxidant activity [46]. Copper is an essential element in enzymatic reactions associated with the wound-healing process. It serves as a cofactor for lysyl oxidase and superoxide dismutase, enzymes essential for collagen production and the prevention of free radical damage. Research has demonstrated that the tripeptide glycyl–histidyl–lysine (GHK), a fragment derived from the alpha chain of collagen, exhibits a high affinity for copper ions, forming a stable GHK–Cu complex. Metals are challenging to transport and often exhibit instability in various pharmaceutical formulations; however, the utilization of carrier peptides has proven to be an effective solution [14].

#### 3.2.1. Copper Tripeptide-1

One of the most used peptides in the cosmetic industry is Copper Tripeptide-1 (GHK–Cu), a hydrophilic molecule consisting of three amino acids: glycine; histidine; and lysine. Its unique properties result from the peptide’s ability to bind copper. Copper Tripeptide-1 is involved in multiple physiological functions of the skin. It can modulate matrix metalloproteinase expression, enzymes responsible for degrading the components of the extracellular matrix, leading to faster healing time of harmed tissue [52]. Also, GHK–Cu stimulates the production of collagen, elastin, and glycosaminoglycan, supporting the growth and functions of dermal fibroblast [53]. Moreover, in combination with hyaluronic acid, it has been demonstrated that Cooper Tripeptide-1 can have a positive impact on collagen IV synthesis [54].

Various studies have demonstrated that Copper Tripeptide-1 can improve the appearance of the skin by reducing the depth of wrinkles, tightening loose skin, protecting cells from UV radiation, stimulating wound healing, and reducing inflammation [55]. Badenhorst et al. have shown that after 8 weeks of applying a serum formulation containing GHK–Cu, the wrinkle depth became significantly smaller compared to the vehicle alone or a commercial product containing Matrixyl 3000 [56]. Huang et al. proved that GHK–Cu used together with light therapy led to an increase in collagen type I and fibroblast growth factor production [57].

#### 3.2.2. Manganese Tripeptide-1

Manganese Tripeptide-1 is a peptide used to carry manganese through the layers of the skin, a metal that is essential for the well-functioning of manganese–superoxide dismutase. This enzyme is responsible for the neutralization of free radicals induced by UV radiation, which can lead to premature aging and tissue damage [58]. Similarly, it is believed that manganese can work as an antioxidant agent to protect the skin from UV-induced degradation. Hussain et al. conducted a study on 15 female participants (40–70 years) presenting moderate photodamage and hyperpigmentation marks. The treatment with Manganese Tripeptide-1 produced a significant reduction, mostly in hyperpigmentation signs, with moderate influence on fine lines and wrinkles. These results conclude that Manganese Tripeptide-1 could be used as an adjoining treatment for hyperpigmentation associated with photoaging [59].

### 3.3. Neurotransmitter Inhibitor Peptides

**Figure 3:**
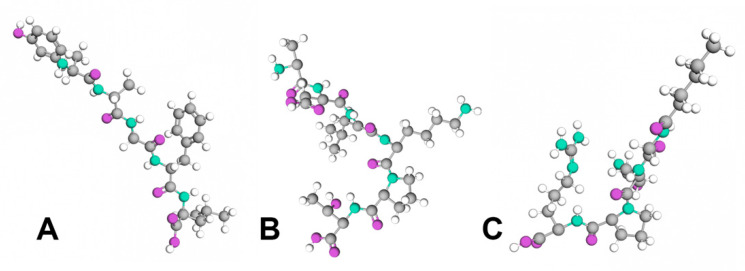
Neurotransmitter inhibitor peptides: (**A**) Pentapeptide-18; (**B**) Acetyl Hexapeptide-3; (**C**) Tripeptide-3.

One of the main causes of wrinkle formation can be sustained muscle contraction, both voluntarily and involuntarily. Acetylcholine (ACh) is the neurotransmitter involved in this process. Muscle contraction occurs when acetylcholine, a neurotransmitter of the parasympathetic nervous system, is released at neuromuscular junctions. This process involves vesicles containing ACh being captured by SNARE (soluble N-ethylmaleimide-sensitive factor activating protein receptor) complexes, which facilitate their fusion with the neuron membrane, allowing ACh to be released into the neuromuscular junction [13]. Once released in the synaptic cleft, it interacts with complexes, binds to the receptor, and generates muscle contraction. This entire process is thought to be controlled by SNAP-25, a receptor protein present in the neuronal membrane, which is associated with the vesicle and directly regulates binding with the SNARE complex as well as the fusion with the membrane of the vesicle. Based on the contraction mechanism, several peptides have been designed with sequences resembling SNAP-25 proteins. These peptides can compete for the binding sites on SNARE complexes, disrupting their structural stability and inhibiting the release of acetylcholine at the neuromuscular junction, ultimately inducing muscle relaxation. The peptides that can prevent muscle cramps and can exhibit specific neuro-suppressive properties are called neurotransmitter inhibitor peptides (Figure 3) [60].

#### 3.3.1. Acetyl Hexapeptide-3

Acetyl Hexapeptide-3 (Argireline^®^) is a synthetic peptide whose amino acid sequence resembles the N-terminal end of the SNAP-25 peptide. The competition with the SNAP-25 destabilizes the SNARE complex and inhibits acetylcholine exocytosis. As a result, Acetyl Hexapeptide-3 can reduce the expression of facial lines and wrinkles by preventing intrinsic muscle contraction. It can be considered an alternative option for botulinic products [61,62]. Since Argireline^®^ is one of the most used cosmetic peptides, new delivery routes and methods of application have been studied to facilitate better permeation through the layers of the skin [63].

Wang et al. have studied the safety and efficacy of Argireline^®^ in the treatment of periorbital wrinkles in Chinese subjects. This randomized, double-blind trial lasted four weeks; then, evaluations were made to determine the efficiency of the active substance. It was concluded that the product containing Acetyl Hexapeptide-3 had a 48.8% anti-wrinkle efficacy compared to the placebo. Analysis of the mechanism of action revealed that Acetyl Hexapeptide-3 has a potency similar to botulinum neurotoxins but with fewer side effects [64]. Blanes-Mira et al. have revealed that after 30 days of treatment with an oil-in-water emulsion containing 10% Argireline, the depth of wrinkles was reduced by up to 30% [65].

#### 3.3.2. Pentapeptide-18

Pentapeptide-18 (Leuphasyl^®^) is a less studied neurotransmitter inhibitor peptide with promising properties. It decreases neuronal activity and neurotransmitter release due to a similar mechanism of action with endogenous enkephalins [66]. In addition to its wrinkle-reduction properties, it has been observed that the inclusion of D-tyrosine in the structure of this botox-like peptide provides the ability to reduce melanin content and tyrosinase activity in melanocytes [67]. Dragomirescu et al. have evaluated the optimal concentration of Pentapeptide-18 for skin application, as well as its safety and effectiveness. They have found that the minimal efficient concentration is 2%, free of side effects, with visible wrinkle reduction. Although the results were beneficial, they proposed combining Argireline^®^ and Leuphasyl^®^ because of their different mechanisms of action, resulting in a more long-lasting effect [25].

#### 3.3.3. Tripeptide-3

Tripeptide-3 (Syn^®^-Ake) is an acetylcholine receptor antagonist similar to the viper venom protein, waglerin-1 [19,68]. Syn^®^-Ake reduces the movement of facial muscles, which ultimately leads to expression wrinkles, mimicking a botox-like activity. This leads to a smoother and even skin surface. Campiche et al. have found a significant decrease in wrinkle parameters after using a formulation containing 4% Tripeptide-10, but no clear improvement was found for smile lines [69]. A study of 45 subjects revealed that Tripeptide-3 improved significantly, by up to 52%, the fine lines of the forehead skin area. Nevertheless, further studies are needed to evaluate the efficiency and safety of Syn^®^-Ake for a better understanding of the way this peptide can be incorporated into cosmetic products [70].

### 3.4. Enzyme Inhibitor Peptides

Enzyme inhibitor peptides prevent the degradation of collagen fibrils and other proteins by inhibiting enzymes responsible for this process, such as superoxide dismutase, stimulate hyaluronan synthase-2, and proteinases. Enzyme-inhibiting peptides function by diminishing the activity of enzymes, particularly matrix metalloproteinases (MMPs), that accelerate skin aging. Increased ROS production from light exposure stimulates MMP synthesis, resulting in excessive degradation of extracellular matrix proteins and a subsequent loss of skin elasticity and firmness. These peptides mimic the enzyme’s natural substrates, bind to the active site, or interfere with enzyme–substrate interactions [34]. This type of cosmetic peptide shows promising results, but not enough clinical studies are available to confirm their benefits in anti-aging products [12]. Some of the most encountered examples, such as soybean, silk, and rice peptides, will be further discussed below.

#### 3.4.1. Soybean Peptides

Soy oligopeptides are obtained by hydrolysis of soybean proteins, consisting of three–six amino acids. Recent studies have shown that soybean peptides exhibit various functions: antioxidant effect; the ability to increase type I collagen; and reduce UV photodamage [71]. Zhou et al. have studied the impact of soybean peptides with no impact on hyperpigmentation spots and hydration while observing a reduction in UVB-induced erythema. Soybean oligopeptides increased the expression of Bcl-2 protein and reduced the level of p53 and Bax proteins. Therefore, soybean peptides can have a positive impact on human skin, offering protection against UV radiation [29,72].

#### 3.4.2. Silk Peptides

Silk peptides are usually extracted from the silk gland of the silkworm *Bombyx mori.* These peptides stand out due to their unique antioxidant and anti-inflammatory properties. Sericin, one of the most important silk peptides, stabilizes free radicals responsible for oxidative damage, capturing 80% of ROS. Its antioxidant potential is strictly correlated to the presence of amino acids rich in hydroxyl groups [73]. Mumtaz et al. have demonstrated that silk proteins possess anti-aging, antioxidant, and antibacterial properties, having the ability to enhance the activity of antioxidant enzymes [74]. In addition, it inhibits lipid peroxidation tyrosinase activity and keratinocyte apoptosis [70].

#### 3.4.3. Rice Peptides

Rice-derived peptides inhibit the activity of metalloproteinases and stimulate the synthesis of hyaluronan synthase 2 genes in keratinocyte cells [13]. Rice protein hydrolysates can exhibit many benefits for the human organism, such as the inhibition of angiotensin I-converting enzyme and antioxidant activity. Therefore, they can be used as an alternative therapy for skin care [75]. In a study conducted by Zhang et al., the results have shown that rice peptides could reduce melanin content (improving hyperpigmentation spots), tyrosinase activity, and ROS levels induced by UV radiation [76].

Cosmetic peptides can be tailored to fit diverse formulations, enabling their integration into a wide array of skincare products. A market study has shown that the interest in incorporating two or more peptides in one formulation has increased in recent times. Moreover, the diversity of peptides almost doubled in the period of analysis, reflecting the growing importance of these ingredients [77]. A summary of different cosmetic formulations containing peptides and the concentrations used can be found in Table 2.

## 4. Limitations of Peptides

The unique properties of cosmetic peptides make them ideal candidates for anti-aging therapy. Recent studies focus not only on their bioactivity but also on the bioavailability and stability of formulations [79]. There are numerous advantages to using peptides in cosmetic products, but developing novel formulations faces challenges and limitations in terms of stability, solubility, and permeation [13].

Another challenge the scientists have faced in the manufacturing processes is maintaining the structural integrity and bioactivity of peptides. These attributes can be affected by the interaction with other components, pH, temperature, and formulation processes (encapsulation, packaging, concentration, and delivery). To overcome this limitation, special attention must be paid when choosing chemically inert or less reactive excipients [7]. In addition, the acquisition of peptides requires increased costs, which depend on the amino acid composition and chain length [31].

Probably one of the most significant disadvantages when referring to peptides is their poor membrane permeability. This property is correlated to the number of hydrogen bonds in the molecule and is favored by their absence. Since peptides possess multiple amide bonds, high hydrophilicity, and, in most cases, large molecular weight, they present difficulties in diffusion through the epithelium [80]. The stratum corneum is very lipophilic and serves as a barrier to hydrophilic compounds, such as peptides. As a result, small molecules diffuse more easily through the layers of the skin [81]. The permeability of peptides through the skin depends on various factors such as the peptide’s physicochemical properties, integrity, thickness of the stratum corneum, area of application, duration of contact, and pharmaco-technical properties of the formulation. For better permeability, molecules should have a molecular weight of less than 500 Da, log of partition coefficient octanol/water between 1 and 3, no or few polar centers, moderate solubility (over 1 mg/mL), and melting point under 200 °C [70]. Additionally, positively charged peptides can facilitate better permeation through membranes as opposed to negatively charged ones. In the context of biological systems where membranes often carry a net negative charge, they interact strongly with these anionic charges, promoting adhesion to the membrane [82]. In particular, the foremost challenge is delivery to the target since most oligopeptides with high molecular weight and low lipophilicity are described as poor in osmosis [13]. Various methods to enhance peptide penetration have been developed, such as chemical modification to form a conjugate with increased lipophilicity, encapsulation into lipophilic carriers, and penetration enhancers [83].

When referring to their physicochemical properties, another limitation is their poor solubility at high concentrations. Some bioactive peptides possess hydrophobic properties and demonstrate low aqueous solubility at high concentrations. To address this challenge, products can be formulated with multiple peptides at lower concentrations, improving solubility while maintaining the desired level of bioactivity. Their solubility depends on the physical properties of the amino acids and the length of the chain, directly influencing the permeability through the epithelium [7].

Moreover, cosmetic peptides can face incompatibilities with various ingredients or environmental factors, impacting their stability, efficacy, and safety. Peptides are molecules composed of many peptide bonds, susceptible to degradation by enzymes such as proteases, which are largely distributed in the whole skin or within formulations, which can reduce their efficacy. Intrinsic factors, like aging, and extrinsic factors, such as UV exposure, pollution, and extreme weather conditions, can lead to heightened enzymatic activity in the skin and peptide degradation [84]. Peptide incompatibilities can result in degradation, reduced efficacy, or altered bioactivity. The effectiveness of cosmetic peptides can vary significantly due to several factors. These include the specific peptide sequence or the properties of the amino acids, which determine their bioactivity, stability, and interaction with target skin cells [85].

Furthermore, their molecular size and structure influence their ability to penetrate the skin barrier and reach the intended target site. Environmental factors, such as pH, temperature, and interactions with other ingredients in the formulation, can also impact their stability and functionality [12]. As a result, the development of peptide-based cosmetics requires careful consideration of these variables to ensure consistent and reliable performance in delivering the desired skin benefits. Most bioactivity studies are conducted using in vitro assays, as these provide a cost-effective method for assessing preliminary biological activity. Moreover, the variable effectiveness associated with peptides may be connected to the inclusion/exclusion criteria of the study’s participants: skin phenotype, age, wrinkle depth, photo aging signs, dermatological disorders, previous invasive, non-invasive, or topical treatments [86]. However, there remains a significant shortage of in vivo clinical trials to confirm the efficacy of bioactive peptides with potential benefits in the cosmeceutical industry [7].

## 5. Methods to Improve Peptide Dermal Delivery

Different methods have been developed to overcome these limitations and facilitate the transport of peptides.

### 5.1. Chemical Penetration Enhancers

Chemical penetration enhancers (CPE) are inactive compounds that can integrate into the stratum corneum, alter its properties, and, thereby, improve drug permeation. The main mechanism of action of chemical enhancers is based on the disruption of the lipid layer of the stratum corneum and the interaction with lipids, proteins, and keratinocytes [87]. In addition, these substances improve drug partition through the skin. An ideal chemical penetration enhancer should be inexpensive, compatible with the peptide, non-toxic, and biocompatible [88]. Propylene glycol, oleic acid, water, and surfactants improve the absorption of active ingredients in cosmetics. Other chemical enhancers, such as fatty acids, sulfoxide, and nitrone, interfere with the lipid layers’ organization and increase transcutaneous penetration. A notable disadvantage is their tendency to irritate the skin after prolonged use [89]. The classification of CPE is mostly based on their chemical structure since many of them exhibit mixed mechanisms of action, as presented in Table 3 [90,91,92,93].

**Figure 4:**
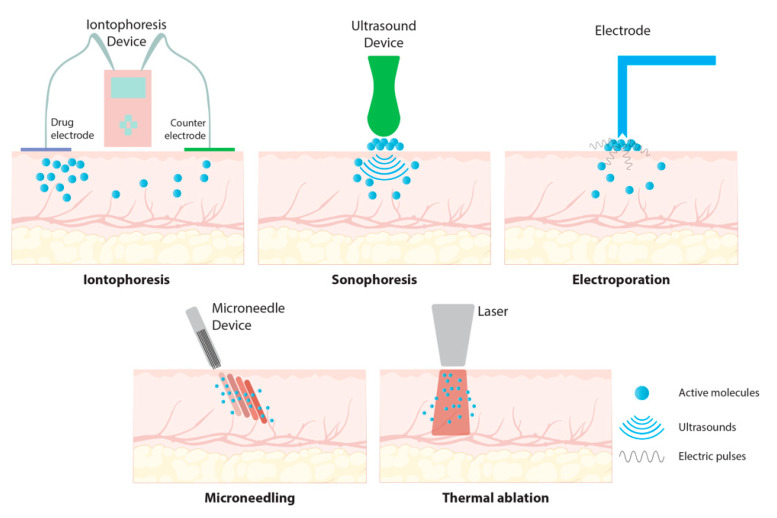
Physical methods used to enhance permeation.

In addition to chemical enhancers, physical methods such as electroporation, microneedles, iontophoresis, ultrasound, and thermal ablation can increase the permeation through the stratum corneum Figure 4 [101].

### 5.2. Electroporation

Electroporation uses high-voltage impulses that are applied to the skin for short periods of time. As a result, aqueous pores are created in the lipid bilayer of the skin, facilitating the transport of both small and large molecules (e.g., DNA, antibodies, peptides, proteins) [105,106]. The drug delivery can be controlled by duration, amplitude, and the number of pulses. Electroporation is often used in combination with other techniques to improve transmembrane transport and topical administration [105]. This method stands out through the advantages it possesses: the parameters can be adjusted to control the permeation rate; the pores created after the high-voltage impulses are reversible; the damage at the skin level is minor, and nonetheless, most drugs that penetrate the skin can be improved (macromolecules, water-soluble substances, charged molecules). Considering this, electroporation has garnered significant attention as an effective technique [107]. However, the above-mentioned method presents a series of limitations, such as inconsistent drug delivery quantities, limited understanding of the underlying mechanisms, and an unpractical electrode configuration [108]. Studies have shown that electroporation can be used for the transport of peptides, but further research is needed to determine the efficiency of this method for cosmetic purposes [109,110].

### 5.3. Iontophoresis

Iontophoresis is a non-invasive technique that uses a low level of electrical current (<500 microA/cm^2^) to transport molecules into the skin when placed under an electrode of the same polarity [111]. The iontophoresis system is comprised of a positive electrode anode, a negative electrode cathode, a drug reservoir, an electronic controller, and a power source. This principle is based on two mechanisms of action: electromigration and electro-osmosis. Firstly, charged molecules move under the influence of an electrical field while in contact with an electrode of the same polarity. This process is called electromigration. The movement is followed by the flow of water, named electro-osmosis, which can transport neutral molecules in the same direction [112]. Iontophoresis is mainly influenced by the applied current, the drug concentration and molecular size, the electrode type, and the pH of the donor [113]. Although it has many advantages, it may exhibit several side effects on the skin: irritation; erythema; and itching [114]. Cosmetic iontophoresis is one of the most evolved technologies used for dermal delivery to intensify the desired effect of the active ingredient. The method targets the epidermis and dermis, layers that are responsible for hydration, texture, firmness, regenerative properties, or pigmentation [115]. Krishnan et al. have studied the effect of iontophoretic techniques and the factors that influence the process of peptide dermal delivery. Alanine–tryptophan dipeptide, acetyl hexapeptide-8, alanine alanine proline valine tetrapeptide, and decapeptide were analyzed under an iontophoresis current of 0.4 mA. The conclusions show that iontophoresis is an efficient method for the dermal delivery of peptides, but several parameters must be optimized to obtain improved results. This study showed that the dermal delivery of peptides is a complex process and is mainly affected by molecular weight, charge, donor pH, donor drug concentration, background electrolytes, and direction of the iontophoresis. It was observed that electroosmosis significantly impacted the permeation of higher molecular-weight peptides, whereas lower molecular-weight peptides were more influenced by electromigration [116].

### 5.4. Microneedles

Microneedle (MN) array is another alternative approach for the physical permeation enhancement. They can penetrate the stratum corneum and create microchannels for the active ingredients to find their way into the deepest layers of the skin without causing permanent damage [117]. Microneedles have very small dimensions ranging from hundred microns in diameter, from units to tens of microns in size of tip and length of 25–2000 µm. They are painless, safe, efficient, easy to use, and can ensure a determined dose delivery. There are four types of microneedles: solid; coated; dissolving; and hollow microneedles [118].

- Solid microneedles are typically used for pretreatment of the skin to create microchannels, after which they are removed, and a patch or another formulation containing the active ingredient is applied. This two-step method is known as “poke and patch” [119]. They deliver the substance through a passive diffusion to skin layers. They can be fabricated from silicon, polylactic acid, or stainless steel [120].
- Coated microneedles are a type of solid microneedles that are coated with active ingredients. This can be achieved by immersing the tip of the needle in a coating solution or by spraying the active content with an atomizer [121];
- Dissolving microneedles are usually obtained by encapsulating the drug into the polymer component of the needle. After penetrating the skin layer, the polymer tip dissolves completely and releases the incorporated substance. This particular type overcomes several problems encountered in the case of other microneedles, such as reducing the risk of injuries [122]. They are made from biocompatible and water-soluble materials (e.g., maltose, polyvinylpyrrolidone, hyaluronic acid, albumin) [123];
- Hollow microneedles have very small channels that carry the drug from a separate reservoir to the desired release site. They are able to hold large quantities of substances and are often equipped with a micro-pump or a syringe to facilitate a controlled drug delivery system. Since only liquid ingredients can be used with this type of needle, they are less used for dermal delivery [124,125].

This delivery method can be influenced by skin physiology, physicochemical properties, and environmental conditions (temperature and humidity). Low humidity can prevent the release of the substance into the skin; excess perspiration can impede microneedle patch adhesion, and very low or very high pH values can affect the permeability of the substance through the stratum corneum [126]. Mohammed et al. have demonstrated that microneedles are an efficient way to improve the dermal delivery of peptides, such as palmitoyl pentapeptide-4, with better results for small molecular weight peptides [127].

### 5.5. Sonophoresis

Sonophoresis uses ultrasound waves at different frequencies to enhance skin permeability and facilitate drug delivery. The better effect is achieved at lower frequencies than high-frequency ultrasound (3–16 MHz). Although it has been researched for the past decade, the exact mechanism of sonophoresis is not yet understood [128]. There are a few mechanisms proposed for this method:

- thermal effects produced by the absorption of ultrasounds;
- the emergence and oscillation of gas bubbles that disrupt the lipid bilayer, known as cavitation;
- mechanical effects [129];

Cavitation is based on the growth, movement, and collapse of bubbles under ultrasound influence, which create shock waves, leading to the alteration of the lipid component of the stratum corneum [130]. The intensity and duration of the treatment depend on the thermal effects; therefore, a combination of low-frequency and high-frequency ultrasound, with a synergic effect, has been found to avoid the overheating of the skin [131]. There are two approaches for the delivery of substances through sonophoresis. Firstly, the formulation is applied to the skin, and the ultrasound is performed immediately after. Secondly, the product can be applied on the desired site, left for a short period to start the absorption process, and then the ultrasound can be applied [132]. In a study conducted by Mutalik et al., it has been demonstrated that sonophoresis successfully enhanced the permeation of peptide dendrimers containing arginine, histidine, lysine, and glycine across the epidermic layer of the skin. A significant improvement was noted compared to the passive diffusion, thus paving the path for further application [133].

### 5.6. Laser Thermal Ablation

Laser thermal ablation is another physical method used to improve drug dermal delivery. There are two types of laser conformation: full beam and fractional beam. Full-beam lasers affect the whole area where the laser is applied, resulting in erythema and slowed healing. Fractional lasers damage only partially the targeted area at a specific depth [134]. The main advantages are fast healing and a large diffusion area [135]. The principle of fractional laser involves three mechanisms: direct ablation; photomechanical wave; and photothermal effect. The skin barrier is ablated, and micropores are created, through which molecules are released and delivered to the deeper layers of the skin [136]. This is performed by the deposition of optical energy, which leads to water evaporation and micropore formation. This process depends on the wavelength, tissue thickness, pulse length, energy, and duration of laser exposure [137]. The different types of lasers used for dermal delivery are mentioned in Table 4 [138,139,140,141]. In the study conducted by Lee et al., it was demonstrated that laser treatment promoted peptide delivery of palmitoyl tripeptide-1 in the affected skin but showed fewer promising results in healthy skin. The confocal microscopy showed that peptides penetrated the skin along the microdots created by the fractional Nd:YAG (neodymium-doped yttrium aluminum garnet) and CO_2_ lasers [142].

### 5.7. Radiofrequency Thermal Ablation

In radiofrequency thermal ablation, metallic microelectrodes similar to needles are placed directly on the skin, and a high-frequency current is applied (100–500 kHz) to create micro-scale pathways. The exposure to radiofrequency causes ionic vibrations in the tissue, localized heat, water evaporation, and, in the end, ablation of cells [143]. Considering the size and density of the microchannels, the rate of drug delivery can be controlled. This method can enhance the permeation of hydrophilic macromolecules in a low-cost and easy way [144]. Its many advantages include the following:

- formation of micro-channels in milliseconds;
- the size and density of the micro-channels can be controlled;
- use for the delivery of a wide range of substances [135].

Xiang et al. studied the dermal delivery of acetyl hexapeptide-8 and palmitoyl pentapeptide-4 using radiofrequency technologies in a liposome formulation. The results showed notably effective synergic effects regarding collagen formation [145].

### 5.8. Dermal Delivery Using Nanosystems

Another method of delivering peptides through the skin’s uppermost layer is using appropriate topical systems. Topical vehicles serve as carrier systems that facilitate the delivery of active molecules across the stratum corneum and deeper into the epidermis and dermis, diminishing the systemic absorption of these substances. The most commonly used topical vehicles are ointments, creams, gels, and lotions. The selection of a topical carrier system depends on factors such as the characteristics of the skin area to be treated and its size to ensure optimal application and contact of the product with the skin [146]. Classical topical formulations can be classified based on their viscosity, as either liquid or semisolid; the number of phases, which may be single-phase or two-phase systems; and the target site of drug action, which may be the skin itself (dermal) or another part of the body following systemic absorption (transdermal) [147].

One of the most promising dermal delivery systems that have gained a lot of interest in the cosmetical industry is nano-systems. This way, compounds are easier to deliver to the deeper layers of the skin. Nano-systems can significantly improve product effectiveness, enabling lower ingredient concentrations, thus reducing skin irritation and delivering higher amounts of active substances [148]. A few examples of nano-systems, their characteristics, and the cosmetic peptides incorporated in these types of formulations are listed in Table 5.

#### 5.8.1. Nanoemulsions

Nanoemulsions are colloidal dispersions comprised of two liquid phases, an aqueous and an oil phase, in which one is dispersed in the other with the help of a surfactant mixture, forming droplets between 20 and 200 nm [160]. They form isotropic, transparent, heterogeneous, and kinetically stable systems, manifesting flocculation and opalescence in time [161]. Nanoemulsions can be oil-in-water (O/W), where the lipophilic phase is dispersed in water, or water-in-oil (W/O), where small particles of water are dispersed in oil. Nevertheless, to add more complexity to this type of formulation, a double emulsion can be formed by obtaining water-in-oil-in-water (W/O/W) or oil-in-water-in-oil (O/W/O) emulsions. They present additional challenges in terms of stability due to being prone to degradation [162]. Nanoemulsion formulations stand out because they present good stability, weak light scattering, unique rheological properties, and the ability to increase the bioavailability of different types of compounds [163]. Samson et al. have studied the influence of a nanoemulsion on the permeability parameters of a copper peptide. The proposed formulation increased the permeability of the peptide through the skin compared to an aqueous solution, which cannot transport the active ingredient with or without a penetration enhancer. This study has proved that nanoemulsions are a viable option for the dermal delivery of peptides [164].

#### 5.8.2. Liposomes

Liposomes are spherical vehicles that can have one or more bilayers (unilamellar, multilamellar) of phospholipids surrounding an aqueous center, with a diameter between 0.05 and 5 μm. They can be obtained from natural or synthetic-derived phospholipids, cholesterol, and polyethylene glycol-derived phospholipids [165]. The use of liposomes in nano-cosmetology offers numerous advantages, such as enhanced penetration and distribution of active ingredients, efficient delivery of active components, prolonged release time, increased stability of active ingredients, reduced unwanted side effects, and high biocompatibility [166]. The study conducted by Dymek et al. has demonstrated that liposomes can be used to incorporate peptides, such as GHK–Cu, in anti-aging cosmetic formulation [52]. Over time, it has been observed that conventional liposomes fail to penetrate the deeper layers of the skin, concentrating in the stratum corneum, as a result of their low potential to deform. This has led to the development of elastic/ultra-deformable liposomes to overcome the limitation. Such examples are ethosomes, niosomes, and transferosomes [167].

#### 5.8.3. Ethosomes

Ethosomes are similar vesicles to liposomes, but they differ because they contain high amounts of ethanol. They are biodegradable, non-toxic, innovative, and biocompatible compounds that enable superior substance delivery [168]. The proposed mechanism for ethosome action is based on the interaction between ethosomes and skin lipids. Firstly, ethanol interacts with the polar head of the endogenous phospholipids, which increases the fluidity and reduces the consistency of the lipid layers. This enables the ethosomes to penetrate the skin with much more flexibility [169,170]. Secondly, “the ethosome effect” confers malleability and fusion with skin lipids, thus releasing the substance in the profound layers [171]. With this type of delivery system, peptides and proteins can be incorporated and delivered. The ethosomal system is a non-invasive method that ensures patient compliance because, in general, it is applied in gel or cream semi-solid form [172]. Nonetheless, they may produce skin irritation due to excipients and can imply higher costs of manufacturing [173]. Kim et al. developed transformer ethosomes composed of phosphatidylcholine and fatty acids containing palmitoyl pentapeptide-4 to enhance skin permeation. The results obtained from the in vitro skin permeation tests showed promising results, indicating that ethosomes can be used as nanocarriers for peptide delivery [154].

#### 5.8.4. Niosomes

Niosomes are versatile delivery systems that are mainly comprised of non-ionic surfactants, cholesterol, or other amphiphilic molecules. Non-ionic surfactants present various roles, such as solubilizers, humectants, and permeability enhancers; thus, their use offers an advantage. In addition, cholesterol influences the fluidity and permeability of the lipidic bilayer [34]. The proposed mechanism is their fusion and interaction with skin’s lipids, combined with modified thermodynamic activity [174]. They have been widely researched for topical delivery because of their ability to enhance penetration through the skin and increase accumulation in the dermis [175]. Niosomes can carry both hydrophilic and hydrophobic molecules, are more stable and less expensive compared to liposomes, and are biodegradable, biocompatible, and non-toxic, representing a safer alternative [176]. Their advantages compared to other nanocarriers make them perfect candidates for peptide incorporation [177]. A study conducted by Badenhorst et al. was performed to assess the suitability of lipid-based systems to enhance the delivery of GHK, particularly niosomes. Their method assessed the stability of GHK–Cu when exposed to all excipients throughout formulation and storage, highlighting the chemical compatibility between the lipids and the peptides encapsulated within the vesicles [155].

#### 5.8.5. Solid Lipid Nanoparticles

Solid lipid nanoparticles (SNLs) are colloidal particles, ranging between 10 and 1000 nm, in which the active substance is dissolved/ encapsulated in lipids. The main components they possess are lipids, surfactants, and co-surfactants. They have been developed to improve the solubility and bioavailability of both hydrophilic and lipophilic ingredients [178]. SNLs are spherical nanoparticles that have a lipid matrix containing the active ingredient and a surfactant surrounding layer to stabilize the system. During storage, they tend to change their conformation and suffer a crystallization process, which may lead to drug expulsion [179]. The advantages of SNLs are presented in Table 6 [180,181,182]. The efficiency of transepidermal transport using solid lipid nanoparticles incorporated with pentapeptide-18 was confirmed by a study performed by Pawłowska et al., in which they tested two cosmetic preparations differing only in the presence of SNLs. Application tests were carried out on females showing signs of aging in the periocular area. After 8 weeks of daily application, the results indicated an improvement in the appearance of the skin and a reduction in wrinkles [156].

#### 5.8.6. Nanostructured Lipid Carriers

Nanostructured lipid carriers (NLC) are second-generation lipid nanocarriers developed to overcome the limitations of SNLs. They have a composition of solid and liquid lipids (70:30) and an aqueous surfactant. The liquid lipids incorporated in the matrix prevent the formation of an ordered crystalline lattice found in the SNL [183]. Because NLCs have an unorganized matrix, there is more space for the active substance, increasing the drug-loading capacity and preventing their expulsion [184]. There are three types of nanostructured lipid carriers. In the imperfect crystal type, solid lipids are mixed with liquid lipids, varying the chain length of fatty acids to maintain an unordered structure [185]. The second type (multiple-type) contains a high quantity of liquid lipids in which the active ingredient is incorporated, forming a nano oil-based compartment. The third type (amorphous model) is obtained by mixing the solid and liquid lipids in a specific way to avoid crystallization and maintain an amorphous state, minimizing drug leakage [186]. When used for cosmetic dermal delivery, they possess the advantage of preventing transepidermal water loss, improving hydration, and facilitating occlusion and emollience [185]. Considering this, NLCs have proved to be innovative and promising carriers for dermal delivery. In their paper, Zielinska et al. have given an overview of NLC products on the market, among which a reconstructive cream with acetyl hexapeptide-3 is included [157].

#### 5.8.7. Lipid Nanocapsules

Lipid nanocapsules (LNC) are a novel carrier system developed as an alternative to conventional delivery methods. They consist of a lipid matrix, in which the active substance can be found, surrounded by capsules of hydrophilic and lipophilic surfactants. By altering the composition, the properties of LNCs can be changed, facilitating the incorporation of a wide range of substances [187]. Initially, they were developed to enhance the delivery of lipophilic drugs, but thanks to their size and composition, hydrophilic substances can also be included [188]. Lipid nanocapsules can permeate the skin by loosening the structure of the lipid bilayer due to fluidization, polarity changes, and lipid exchange. LNCs present significant potential for dermal and topical application as a result of their small size, high encapsulation efficiency, biocompatibility, and stability of encapsulated substances [189]. Lipotec has developed a new delivery system based on polymeric nanocapsules that contain microemulsions of water in oil, which can incorporate peptides such as GHK, Tripeptide-10 Citrulline, or Pentapeptide-4 [158].

#### 5.8.8. Nanospheres

Nanospheres are used in cosmetic preparations to deliver active ingredients to the deeper layers of the skin, exhibiting anti-wrinkle, hydrating, and anti-acne effects [190]. They differ from nanocapsules in terms of composition, with nanospheres having a polymeric matrix system. The active substance can be dissolved in the matrix or adsorbed on the polymeric surface [191]. This type of system was used to incorporate acetyl octapeptide-3 to find new methods for anti-wrinkle therapy and can be used as an alternative drug release system to Botox treatment [26].

#### 5.8.9. Liquid Crystalline Nanoparticles

Liquid crystalline nanoparticles are an emerging class of nanomaterials developed for enhanced drug delivery. Their properties can be adjusted to accommodate a large number of ingredients: peptides; proteins; and nucleic acids [192]. They can be divided into thermotropic and lyotropic crystals, the latter being used for drug delivery. Lyotropic liquid crystals are amphiphilic molecules existing in mesophases that can undergo phase transition depending on the water content, resulting in hexagonal, cubic, and lamellar phases. In addition to the aqueous component, they contain a mixture of surfactants [193]. They present a few advantages over the existing nanocarriers: multi-drug loading; controlled drug release; reduced toxicity due to the use of biocompatible lipids; and improved permeation of macromolecules such as peptides [194]. Akhlaghi et al. have studied the stability and release profile of cubosomes loaded with palmitoyl-GHK and palmitoyl tetrapeptide-7, the results suggesting that these formulations can have applications in the development of topical anti-aging systems [159].

## 6. Conclusions

In conclusion, peptides are promising active ingredients that have raised the interest of many scientists in the cosmetic industry due to their unique anti-aging properties. The mentioned studies demonstrate their efficacy in products used to improve the appearance of aging signs and highlight the need for further research to achieve their full potential. Cosmetic peptides have various mechanisms of action that allow them to modulate intracellular pathways, thus also preventing the development of new aging signs such as wrinkles, fine lines, loss of firmness, and elasticity. Despite the numerous advantages that recommend peptides for anti-aging use, their shortcomings often limit their application. The advances in product formulation have facilitated ways to overcome their limitations regarding membrane permeability and dermal delivery. Novel nano-formulations are now available to incorporate cosmetic peptides for easier delivery into the dermis. Nevertheless, chemical enhancers and physical methods such as electroporation, microneedles, sonophoresis, and thermal ablation are methods that can be used for better permeation through the stratum corneum. Considering this, cosmetic peptides are active ingredients that can be used to develop innovative products with enhanced permeability through the skin barrier and superior efficiency.

## Figures and Tables

**Table 2:** Cosmetic formulations with peptides.

Peptide	Formulation Type	Concentration	References
Palmitoyl Pentapeptide-4	o/w cream	0.0003%	[49]
Tripeptide-10 Citrulline	w/w cream	5%	[50]
Palmitoyl hexapeptide-12	w/w cream	0.0004%	[34]
Copper Tripeptide-1	serum	1%	[57]
Acetyl Hexapeptide-3	o/w or w/w cream	10%	[50,65]
Pentapeptide-18	o/w cream	2%	[25]
Acetyl Octapeptide-3	solution	0.05%	[78]
Tripeptide-3	o/w cream	4%	[69]

w/w—water in water; o/w—oil in water.

**Table 3:** Main types of chemical penetration enhancers used in peptide formulations.

Class of CPE	Examples	Applications	Mechanism of Action	Advantages	Disadvantages	References
Sulfoxides	Dimethylsulfoxide (DMSO) Dimethylformamide (DMF) Dimethylacetamide (DMAC)	PKEK	Alters the intercellular keratin structure.	Enhanced permeation of hydrophilic as well as hydrophobic drugs.	At high concentrations, it can cause erythema, scaling, contact urticaria, stinging and burning feelings, and produce a malodorous metabolite in the breath.	[94]
Fatty acids	Oleic acid Lauric acid Caprylic acid	Palmitoyl pentapeptide-4, Acetyl hexapeptide-8	Interaction and disruption of the lipid layer. Increases skin diffusion.	Similar hydrophobicity to the lipids in the stratum corneum.	Dermal side effects.	[95,96]
Alcohols	Ethanol Buthanol Propanol Lauryl alcohol	Acetyl hexapeptide-8, Palmitoyl tetrapeptide-7, Palmitoyl tripeptide-1, Dipeptide-2	Solvent increases the solubility of the peptide. Modifies the thermodynamic activity of the drug within the formulation. Disrupts the polar head from the lipid layer.	Rapid permeation.	Short residence in the skin due to its volatility.	[86]
Glycols	Propylene glycol Dipropylene glycol	PKEK, Acetyl hexapeptide-8, Tetrapeptide-68, Rice peptides	Cosolvent Gradient concentration mechanism Improves the partition properties of substances.	Synergic action when used with other CPE, e.g., oleic acid.	Increases transepidermal water loss and protease activity. Reduces the barrier role of the skin.	[81,97,98,99]
Urea		Palmitoyl tripeptide-38	Increases the water content in the skin. Activates keratolytic activity.	Very high solubility in water. Promotes skin rehydration.	Modest penetration-enhancing activity.	[100]
Terpenes, terpenoids and essential oils	Monoterpenes: D-Limonene, Menthol, Cineol, Carvacrol Sesquiterpenes: Farnesol, Neridol, Bisabolol	Decapeptide-12, Palmitoyl pentapeptide-4	Modifies the solvent nature of the stratum corneum. Improves substance partition Disrupts the lipid bilayer.	Less skin irritation polar terpenes improve the permeation of hydrophilic molecules; non-polar terpenes (D-limolene) improve the penetration of hydrophobic molecules.	Volatile compounds.	[101,102]
Surfactants	Anionic: sodium lauryl sulfate Cationic: alkyl dimethylbenzyl ammonium chloride, cetyltrimethylammonium bromide Nonionic: polysorbate 80 (Tween 80), dodecyl betaine	Silk peptides, Palmitoyl tripeptide-8, Pentapeptide-18	Solubilizes the lipids of the skin. Disrupts the lipid and protein domains. Interacts with intercellular keratin.	Amphiphilic molecules and non-ionic surfactants exhibit less irritating side effects. Anionic surfactants present better permeation effects.	Anionic and cationic surfactants are powerful irritants. Increased transepidermal water loss; non-ionic surfactants have only minor enhancement effects in human skin.	[18,103,104]

**Table 4:** Types of lasers.

Laser	Wavelength (nm)	Pulse Length
CO_2_	10,600	0.5–2.5 ps
Erbium:YAG	2940	500 μs
P.L.E.A.S.E. (Precise Lasers Epidermal System)	2940	3 μs

**Table 5:** Characteristics of nano-systems used for peptide dermal drug delivery.

Type	Structure	Diameter	Characteristics	Application	References
Nanoemulsions	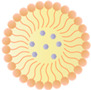	20–200 nm	A colloidal dispersion comprised of two liquid phases and a surfactant.	Tripeptide-3, Cu-GHK	[149,150]
Liposomes	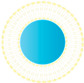	0.05–5 μm	Vesicular system with an aqueous core and a phospholipidic membrane.	Palmitoyl tripeptide-5, Acetyl hexapeptide-3, Palmitoyl Pentapeptide-4	[151,152]
Ethosomes	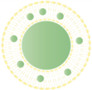	>30 nm	Vesicles with a deformable structure that contain phospholipids and a high concentration of ethanol (45%).	Palmitoyl pentapeptide-4	[153,154]
Niosomes	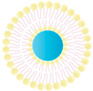	0.1–2 μm	Structures comprised of non-ionic surfactants and cholesterol.	Cu-GHK	[155]
Solid lipid nanoparticles	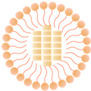	10–1000 nm	Spherical nanoparticles with solid lipids surrounded by a surfactant layer.	Pentapeptide-18	[156]
Nanostructured lipid carriers	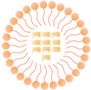	10–1000 nm	Nanoparticles whose matrix is a combination of solid and liquid lipids.	Dipeptide-1, Acetyl hexapeptide-3	[27,157]
Lipid nanocapsules	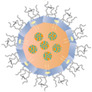	100–500 nm	Structures with a lipidic core and hydrophilic/ hydrophobic surfactants.	GHK	[158]
Nanospheres	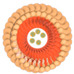	10–200 nm	Spheres that possess a polymeric matrix.	Acetyl octapeptide-3	[26]
Liquid crystalline nanoparticles	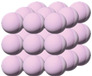	10–500 nm	Molecules that suffer phase transition in certain conditions.	Pal-GHK, palmitoyl tetrapeptide-7	[159]

**Table 6:** The main advantages of solid lipid nanoparticles.

Advantages of SNLs
Controlled and targeted drug release
Strong skin adhesion
Good reproducibility and large-scale production
Improved stability of the active ingredient
Water based technology
Biocompatibility and biodegradability
Lipophilic and hydrophilic molecule incorporation
Affordable compared to other nanocarriers
Increased permeation

## Data Availability

No new data were created or analyzed in this study. Data sharing is not applicable to this article.
